# Genome-Wide Analysis of DEAD-box RNA Helicase Family in Wheat (*Triticum aestivum*) and Functional Identification of *TaDEAD-box57* in Abiotic Stress Responses

**DOI:** 10.3389/fpls.2021.797276

**Published:** 2021-12-09

**Authors:** Jing-Na Ru, Ze-Hao Hou, Lei Zheng, Qi Zhao, Feng-Zhi Wang, Jun Chen, Yong-Bin Zhou, Ming Chen, You-Zhi Ma, Ya-Jun Xi, Zhao-Shi Xu

**Affiliations:** ^1^State Key Laboratory of Crop Stress Biology for Arid Areas, College of Agronomy, Northwest A&F University, Yangling, China; ^2^Institute of Crop Science, Chinese Academy of Agricultural Sciences/National Key Facility for Crop Gene Resources and Genetic Improvement, Key Laboratory of Biology and Genetic Improvement of Triticeae Crops, Ministry of Agriculture, Beijing, China; ^3^Hebei Key Laboratory of Crop Salt-Alkali Stress Tolerance Evaluation and Genetic Improvement/Cangzhou Academy of Agriculture and Forestry Sciences, Cangzhou, China

**Keywords:** DEAD-box, genome-wide analysis, expression profile, abiotic stress response, regulation mechanism, wheat

## Abstract

DEAD-box RNA helicases constitute the largest subfamily of RNA helicase superfamily 2 (SF2), and play crucial roles in plant growth, development, and abiotic stress responses. Wheat is one of the most important cereal crops in worldwide, and abiotic stresses greatly restrict its production. So far, the DEAD-box RNA helicase family has yet to be characterized in wheat. Here, we performed a comprehensive genome-wide analysis of the DEAD-box RNA helicase family in wheat, including phylogenetic relationships, chromosomal distribution, duplication events, and protein motifs. A total of 141 *TaDEAD-box* genes were identified and found to be unevenly distributed across all 21 chromosomes. Whole genome/segmental duplication was identified as the likely main driving factor for expansion of the *TaDEAD-box* family. Expression patterns of the 141 *TaDEAD-box* genes were compared across different tissues and under abiotic stresses to identify genes to be important in growth or stress responses. *TaDEAD-box57-3B* was significantly up-regulated under multiple abiotic stresses, and was therefore selected for further analysis. TaDEAD-box57-3B was localized to the cytoplasm and plasma membrane. Ectopic expression of *TaDEAD-box57-3B* in *Arabidopsis* improved tolerance to drought and salt stress as measured by germination rates, root lengths, fresh weights, and survival rates. Transgenic lines also showed higher levels of proline and chlorophyll and lower levels of malonaldehyde (MDA) than WT plants in response to drought or salt stress. In response to cold stress, the transgenic lines showed significantly better growth and higher survival rates than WT plants. These results indicate that *TaDEAD-box57-3B* may increase tolerance to drought, salt, and cold stress in transgenic plants through regulating the degree of membrane lipid peroxidation. This study provides new insights for understanding evolution and function in the *TaDEAD-box* gene family.

## Introduction

RNA helicases unwind double-stranded RNA in an ATP-dependent manner through hydrolysis (Lorsch, 2002; Gong et al., 2005; Cordin et al., 2006; Linder, 2006; Chen et al., 2014). The DEAD-box RNA helicases constitute the largest subfamily of RNA helicase superfamily 2 (SF2), and are ubiquitous in both prokaryotes and eukaryotes (Mingam et al., 2004; Rocak and Linder, 2004). Within the SF2 superfamily, *DEAD-box*, *DEAH-box*, and *DExD/H-box* families are distinguished by variations in motif II; DEAD-box RNA helicases are named after the highly conserved residues Asp-Glu-Ala-Asp (D-E-A-D) comprising motif II (Caruthers and McKay, 2002; Cordin et al., 2006; Linder and Owttrim, 2009). DEAD-box RNA helicases contain at least nine conserved motifs in the helicase core domain, which are involved in ATPase and helicase activities (Tanner et al., 2003; Banroques et al., 2011; Linder and Jankowsky, 2011; Putnam and Jankowsky, 2013). In addition to the conserved motifs, the N- and C-terminal extensions in the *DEAD-box* family vary in length and composition, and these play roles in substrate binding specificity, helicase activity, subcellular localization, and interaction with components (Cordin et al., 2006; Fairman-Williams et al., 2010; Banroques et al., 2011; Byrd and Raney, 2012).

DEAD-box RNA helicases have been reported to function in all aspects of RNA metabolism (Bowers et al., 2006; Cordin et al., 2006; Linder, 2006; Linder and Fuller-Pace, 2013; Putnam and Jankowsky, 2013; Russell et al., 2014). For example, DEAD-box RNA helicase AtRH57 may play a role in the formation of small ribosomal subunits (Hsu et al., 2014). DEAD-box RNA helicase RCF1 maintains proper splicing of pre-mRNAs (Guan et al., 2013). DEAD-box protein UAP56 interacts with the mRNA export factors ALY2 and MOS11, which are involved in exporting mRNA from the plant cell nucleus (Kammel et al., 2013). UPF2 eliminates aberrant mRNAs that are derived from various sources, which is necessary for the nonsense-mediated mRNA decay process (Shi et al., 2012). RH50 is involved in efficient translation of plastid proteins by comigrating with ribosomal particles, indicating that RH50 may act as a 23S-4.5S rRNA maturation factor, functionally overlapping with the plastid signaling factor GUN1 (Paieri et al., 2018). DEAD-box RNA helicases disrupt misfolded RNA structures, thus acting as chaperones to promote correct RNA folding through unwinding activity or RNA-protein association/dissociation (Lorsch, 2002; Gong et al., 2005; Kant et al., 2007).

DEAD-box RNA helicases have also been reported to participate in plant growth and development (Kant et al., 2007; Shi et al., 2012; Bush et al., 2015, 2016; Huang et al., 2016a,b; Chen D. et al., 2020). ISE2 is involved in posttranscriptional gene silencing and cell fate determination, affecting plasmodesmata structure and function through the regulation of RNA metabolism and subsequent gene expression (Kobayashi et al., 2007). *AtRH7/PRH75*-knockout mutants show auxin-related developmental defect phenotypes that have also been observed in several ribosomal protein mutants, indicating that *AtRH7* may participate in rRNA biogenesis and plant development (Huang et al., 2016a). eIF4A interacts with cyclin-dependent kinase A (CDKA) in proliferating cells and is modulated by phosphorylation, thereby affecting translation. Loss of *eIF4A1* function results in reduced plant growth and dramatically decreased fertility (Bush et al., 2015, 2016). An *rh2*9 mutant was shown to have nonfunctional gametophytes and defective pollen tube growth in the pistil, implying that gametophyte-specific *RH29* may play important roles in functional maturation of male and female gametophytes (Chen D. et al., 2020). Rice *OsRH2* and *OsRH34* were identified as core components of the exon junction complex (EJC) and are involved in regulating plant height and development of pollen and seeds in rice (Huang et al., 2016b).

Increasing evidence has revealed that DEAD-box RNA helicases play crucial roles in plant abiotic stress responses, including drought, salt, temperature, and oxidative stress. A *los4-1* mutation has been shown to reduce cold regulation of C-repeat binding factors (CBFs) and their target genes, and mutants exhibit a chilling-sensitive phenotype. This indicates that *CRYOPHYTE/LOS4* may play an important role in mRNA export and stress responses (Gong et al., 2005). *strs1* and *strs2* mutants have increased tolerance to salt, osmotic, and heat stresses, whereas *STRS*-overexpressing lines display less stress tolerance. STRS proteins are involved in epigenetic silencing of gene expression through re-localization to suppress the stress responses in *Arabidopsis* (Kant et al., 2007; Khan et al., 2014). *AtRH9* and *AtRH25* affect seed germination under salt stress, and different nucleic acid binding properties lead to differences in cold tolerance (Kim et al., 2008). Rice *OsPDH45* mediates salinity stress tolerance by controlling the antioxidant system and protecting the photosynthetic machinery (Gill et al., 2013). The rice nucleolar DEAD-box RNA helicase TOGR1 is regulated by temperature and circadian rhythm, and is associated with the small subunit (SSU) to facilitate effective pre-rRNA processing; it is therefore involved in regulating rice thermotolerant growth as a key pre-rRNA chaperone for rRNA homeostasis (Wang D. et al., 2016). Additionally, OsTOGR1 may be a positive factor for regulation of heat stress tolerance in Chinese cabbage (Yarra and Xue, 2020). *tcd33* displays an albino phenotype and severe defects of chloroplast structure under 20°C condition; however, overexpression of *TCD33* confers cold tolerance by regulating rice chloroplast development and the expression of cold-responsive genes (Wang X. M. et al., 2020).

Genome-wide analyses of the RNA Helicase gene family have been undertaken in *Arabidopsis*, rice, tomato, maize, cotton, and sweet potato (Umate et al., 2010; Xu et al., 2013a,b; Wan et al., 2020). However, few reports exist describing identification and functional analysis of *DEAD-box* genes in wheat. Wheat is one of the most important cereal crops worldwide, serving as a key staple crop for humans (Oono et al., 2013; Marcussen et al., 2014). Severe abiotic stresses limit wheat production (Zhan et al., 2017). Because wheat now has a fully annotated reference genome, studies related to gene function are now more feasible than they were previously (Appels et al., 2018). Identifying stress resistance genes and exploring their functions are important goals towards improving stress responses through wheat molecular breeding. In this study, we revealed that *TaDEAD-box57-3B* improved diverse abiotic stresses resistance in transgenic *Arabidopsis* through regulating the degree of membrane lipid peroxidation. This study provides a basis for better understanding the *TaDEAD-box* gene family and the function of *TaDEAD-box57-3B* in response to abiotic stresses.

## Materials and Methods

### Identification of DEAD-box Genes in Wheat

The identification of wheat *DEAD-box* gene family was performed according to the previous methods described by Yu et al. (2019) with some revisions. Firstly, to identify the *TaDEAD-box* genes in wheat, protein sequences of all *DEAD-box* genes in *Arabidopsis* and rice were downloaded from TAIR - Home Page (TAIR)^1^ and Rice Genome Annotation Project (RGAP) databases^2^ with National Center for Biotechnology Information (NCBI) as a complementary database^3^. Wheat reference sequences were downloaded from the Ensembl Plants database^4^ to build a local protein database. The protein sequences of all *Arabidopsis* and rice were used as queries to perform local BLASTp searches (with *e*-value < 1e-10) against local wheat database. Secondly, the candidate sequences were used to build a wheat-specific HMM profile through hmmbuild program. Then the hmmsearch program was used to obtain all TaDEAD-box candidate members. All candidate proteins were submitted to NCBI Batch CD-search^5^ and Pfam databases^6^ for examining the presence of DEAD-box domain. We identified all candidate sequences with the conserved D-E-A-D motif. After validation, all candidate *TaDEAD-box* genes were identified in the wheat genome (Supplementary Table 1). The molecular weight (MW) and isoelectric point (pI) values were predicted by online tool ExPASy^7^. Subcellular localizations were predicted through online tools BUSCA^8^.

### Multiple Sequence Alignment and Phylogenetic Analysis

Multiple sequence alignment of amino acid sequences was performed using ClustalX, and then optimum model was used to generate the interspecific phylogenetic tree (*Arabidopsis*, rice, wheat) by MEGA X based on the maximum-likelihood (ML) method with 1000 bootstrap replicates to determine the statistical reliability of the phylogenetic trees. In addition, the phylogenetic tree of TaDEAD-box family was built by MEGA X based on neighbor-joining (NJ) method with 1000 bootstrap replicates to identify the consistency of phylogenetic tree.

### Chromosomal Location and Gene Duplication

The wheat annotated reference genome sequences were downloaded from Ensembl Plants database. All *TaDEAD-box* genes were mapped against the 21 wheat chromosomes using positional information by TBtools software (Chen C. et al., 2020). Homoeologous genes were identified through phylogeny tree and Ensembl Plants database (Schilling et al., 2020). Segmental and tandem duplication events were recognized according to the previous study (Wang M. et al., 2016; Fan et al., 2019). Related synteny blocks and duplicated gene pairs were calculated using MCScanX (*e*-value ≤ 1e-10) and visualized using Circos software (Krzywinski et al., 2009). The nonsynonymous substitution rate (Ka), synonymous substitution rate (Ks) and the Ka/Ks ratio between duplicated gene pairs were calculated by TBtools software. Ka/Ks ratio > 1 represents positive selection, Ka/Ks ratio = 1 represents neutrality, and Ka/Ks ratio of < 1 represents purifying selection.

### Gene Structure and Conserved Motifs

The coding sequences and genome sequences of *TaDEAD-box* genes were obtained from the Ensembl Plants database, and the exon/intron structure was visualized by TBtools software (Chen et al., 2018). The online MEME program^9^ with 15 motifs and motif width of 6-50 amino acids was applied to identify the conserved motifs of all members (Bailey et al., 2009). The results were visualized and rearranged by TBtools software (Chen C. et al., 2020).

### *Cis*-Elements Analysis

To analysis the possible biological functions and transcriptional regulation of 141 *TaDEAD-box* genes. The 2.0 kb region sequences upstream from start codons were downloaded from Ensembl Plants database and then submitted to PlantCARE database for *cis*-elements analysis^10^ (Lescot et al., 2002).

### Expression Pattern Analysis by Transcriptomic Data

Transcriptomic data (choulet_URGI) used to analyze the expression patterns of *TaDEAD-box* genes in different tissues and stages were downloaded from Wheat Expression browser^11^ database (Borrill et al., 2016; Ramirez-Gonzalez et al., 2018). Besides, transcriptomic data (SRP045409, SRP043554) were also extracted to study the expression level of *TaDEAD-box* genes under abiotic stresses (Li et al., 2015; Liu et al., 2015). The expression patterns of *TaDEAD-box* genes were estimated using (log_2_TPM+1) (transcripts per million) values through pheatmap package of R project13. The TPM values of *TaDEAD-box* genes in different tissues and under abiotic stresses (cold, drought, high-temperature) were shown in Supplementary Table 7.

### Plant Materials and Stress Treatments

The wheat cultivar Xiaobaimai grown in Hoagland’s liquid medium was used for gene expression patterns analysis. The wild type *Arabidopsis* cultivar Columbia-0 was used as background for transformation and subsequent phenotypic assays. All the plants were grown in a greenhouse with 60% relative humidity, 22°C, and a photoperiod of 16-h light/8-h dark. Seven-day-old wheat seedlings were used for dehydration (250 mM Mannitol), salt (200 mM NaCl), heat (42°C), and cold (4°C) stress treatments. Seedlings were transferred into solutions containing 250 mM Mannitol or 200 mM NaCl for dehydration drought or salt treatment. Seedlings were placed in 42°C or 4°C incubator for heat or cold treatment. The samples were collected at 0, 0.5, 1, 2, 4, 8, 12, 24 h after treatments, and then immediately frozen in liquid nitrogen and stored at -80°C for RNA extraction.

### RNA Isolation and Quantitative Real-Time PCR

Total RNA was extracted from wheat leaves using RNA plant extraction kit (Zhuangmeng, Beijing, China), and *Transcript*^®^ All-in-One First-Strand cDNA Synthesis SuperMix (TransGen Biotech, Beijing, China) was used for reverse transcription following the manufacturer’s instruction. Quantitative Real-Time PCR (qRT-PCR) was performed on an Applied Biosystems 7500 Real-Time PCR System with Super Real PreMix Plus (SYBR Green) (TIANGEN, China). The wheat β*-actin* (GenBank: MF405765.1) was used as an internal control for normalization of the template cDNA. Each experiment was performed with three biological replicates. The quantitative results analysis was performed using the 2^–ΔΔ*CT*^ method (Udvardi et al., 2008). The primers used for qRT-PCR are listed in Supplementary Table 8.

### Subcellular Localization

The full-length coding sequence (CDS) of *TaDEAD-box57-3B* was amplified from wheat cultivar Xiaobaimai and Jinhe991, and no allelic variations were observed. The CDS excluding the termination codons of *TaDEAD-box57-3B* was amplified and cloned into p16318hGFP vector under control of the CaMV35S promoter. For transient expression assays, 7-day-old wheat seedlings were used for the isolation of wheat protoplasts as previously described (Liu P. et al., 2013). The p16318hGFP-*TaDEAD-box57-3B* and control plasmids were transformed into wheat protoplasts mediated by polyethylene glycol 4000 (PEG4000) as described by Liu P. et al. (2013). The transfected protoplasts were incubated at 22°C for 18 h in darkness, after which GFP signals were observed with 488 nm and 543 nm illumination by a confocal laser scanning microscopy (LSM700; CarlZeiss). The primers are listed in Supplementary Table 8.

### Generation of Transgenic *Arabidopsis*

The CDS excluding the termination codons of *TaDEAD-box57-3B* was cloned into plant expression vector pCAMBIA1302 under the control of CaMV35S promoter. After sequencing, the correct plasmid pCAMBIA1302-*TaDEAD-box57-3B* was transformed into *Arabidopsis* by *Agrobacterium tumefaciens*-mediated floral dip method (Clough and Bent, 1998). The transformed seeds were surface sterilized with sodium hypochlorite and selected on 1/2 MS medium containing 50 ug/ml hygromycin and then transferred to soil (rich soil: vermiculite = 1:1). T_3_ generation plants were plants were cultured at 22°C and 60% relative humidity with a photoperiod of 16-h light/8-h dark. Three homozygous T_3_ lines were selected for the following phenotypic analysis.

### The Abiotic Stress Treatments of Transgenic *Arabidopsis*

For the germination assay, T_3_ generation seeds of transgenic lines and WT were grown on Murashige and Skoog (MS) and MS medium supplemented with 300 mM, 400 mM mannitol, 100mM, and 125 mM NaCl. The seeds were kept at 4°C for 3 d to break dormancy, and then transferred to a tissue culture room with a photoperiod of 16-h light/8-h dark at 22°C (60% relative humidity; 100 μmol m^–2^ s^–1^ light intensity). We defined the seed germinated when radicles had emerged from the seed coat. Seed germination was followed for the next 6 days, and the germination rate was calculated as a percentage. For the root growth assay, 5-day-old seedlings were transferred to MS medium with or without 200 mM, 300 mM mannitol, 100mM, and 125 mM NaCl for another 7 days, and then the total root lengths and fresh wights were measured. All experiments contained three independent replicates.

For tolerance tests, the disinfected seeds were planted in MS medium for 7 days, and then transplanted to the soil (1:1 mixture of rich soil and vermiculite) at 22°C with a photoperiod of 16-h light/8-h dark. The drought phenotype analysis was performed as described (Liu W. X. et al., 2013). For drought treatment, 21-day-old seedlings were treated by withdrawing irrigation until there were significant wilting differences between WT and transgenic plants. The survival rates were measured after re-watering for 7 days. For salt treatment, 7-day-old seedlings were transferred to the soil at 22°C with a photoperiod of 16-h light/8-h dark, and then 21-day-old seedlings were irrigated with 200 mM NaCl until significant differences were observed between WT and transgenic plants. The survival rates were recorded and all the assays were repeated three times.

For cold treatment, disinfected seeds were planted in MS medium for 5 days, and then were placed at 4°C for 1 d followed by −8°C for 1 h. After freezing treatment, the plants were transferred to 4°C for 12 h and then resumed normal grown at 22°C for additional 3 days. The survival rates were recorded and all the tests contained three independent replicates.

### Measurements of Chlorophyll, Proline, and Malonaldehyde (MDA) Contents

To better understand the function of *TaDEAD-box57-3B* under abiotic stresses, the physiological indicators (chlorophyll, proline, MDA contents) were measured according to the manuals of the corresponding kit (CPL-1-G, PRO-1-Y, MDA-1-Y, Cominbio, Suzhou, China). After drought treatment for 2 weeks and salt treatment for 1 week, the leaves were mixed and collected for physiological indicator measurement. Absorbance values were measured with a Varioskan LUX Multimode Microplate Reader (Thermo Fisher Scientific). All of the experiments were repeated three times.

### Statistical Analysis

All the experiments contained three independent replicates. Values are means ± standard deviations (SDs) of three replicates, and asterisks (^∗^ or ^∗∗^) represent the significant differences at *p* < 0.05 or *p* < 0.01, respectively (ANOVA test).

## Results

### Identification of *TaDEAD-box* Genes in Wheat

Based on conserved domains and motifs, a total of 141 *TaDEAD-box* candidate genes were identified in the wheat genome (*Triticum aestivum* IWGSC). For convenience, these candidate genes were renamed based on their chromosomal locations, from *TaDEAD-box1-1A* to *TaDEAD-box141-7D*. Detailed information and physicochemical properties of these genes are summarized in Supplementary Table 1. The amino acid sequences ranged from 354 to 1347 in length, with an average of 647. Physicochemical properties varied greatly in the *TaDEAD-box* family. The molecular weight (MW) values ranged from 39.04 kDa to 148.50 kDa, with an average of 71.55 kDa, and isoelectric point (pI) values ranged from 4.92 to 10.17, with 76.6% of members (108/141) exhibiting alkaline pI values.

### Phylogenetic and Homologous Analysis of TaDEAD-box Members

**FIGURE 1:**
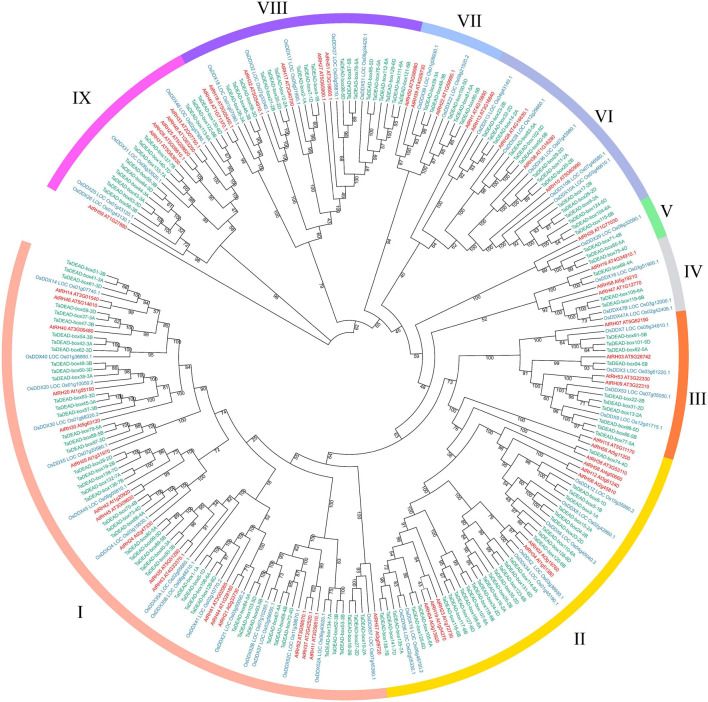
Phylogenetic analysis of DEAD-box proteins in wheat, rice and *Arabidopsis*. A total of 245 proteins were used to construct the maximum-likelihood (ML) phylogenetic tree by MEGA X with 1,000 bootstrap replicates. The DEAD-box proteins were divided into nine groups (I-IX), which were marked with different colors. The members of three species were, respectively, marked with different colors: red for *Arabidopsis*, blue for rice, and green for wheat.

To analyze evolutionary relationships among the 141 TaDEAD-box proteins, phylogenetic analysis was conducted using an additional 57 DEAD-box proteins from *Arabidopsis* and 47 from rice, for a total of 245 genes (Figure 1). A separate phylogenetic tree was also built using only the TaDEAD-box proteins for comparison. Based on amino acid sequence similarities and the phylogenetic analysis from *Arabidopsis*, DEAD-box members were divided into nine groups (I-IX). Group I harbored the most DEAD-box members of the three species, implying that genes in Group I were frequently retained during evolution.

Because there are three homoeologous subgenomes in common wheat (A, B, and D), every wheat gene may be part of a three-homoeolog triad (Yu et al., 2019). We identified homoeologous genes through phylogenetic tree construction and BLAST searches. The results revealed that the 141 *TaDEAD-box* genes represented 141 homoeologs of 55 genes: 38 genes had all three homoeologs, 10 genes had two homoeologs, and seven genes had only one homoeolog (Table 1). Prior research showed that 35.8% of genes in the wheat genome have three homoeologous genes (Appels et al., 2018; Xu et al., 2021), meaning that *TaDEAD-box* homoeologs are retained at a higher rate; 69.1% of *TaDEAD-box* genes vs. 35.8% of genes on average are present in triads, 18.2% vs. 13.2% are present in pairs, and 12.7% vs. 37.1% are present as a single gene. Consequently, the high homoeolog retention rate may explain why the number of *DEAD-box* genes in wheat is much higher than it is in *Arabidopsis* and rice.

**TABLE 1:** Distribution pattern of TaDEAD-box homoeologs.

Distribution pattern		Number of genes
Three homoeologs	A, B, D	38
Two homoeologs	A, B	4
	A, D	4
	B, D	2
One homoeolog	A	3
	B	3
	D	1
Total homoeologs	141	55

### Chromosomal Location and Duplication Events of *TaDEAD-box* Genes

**FIGURE 2:**
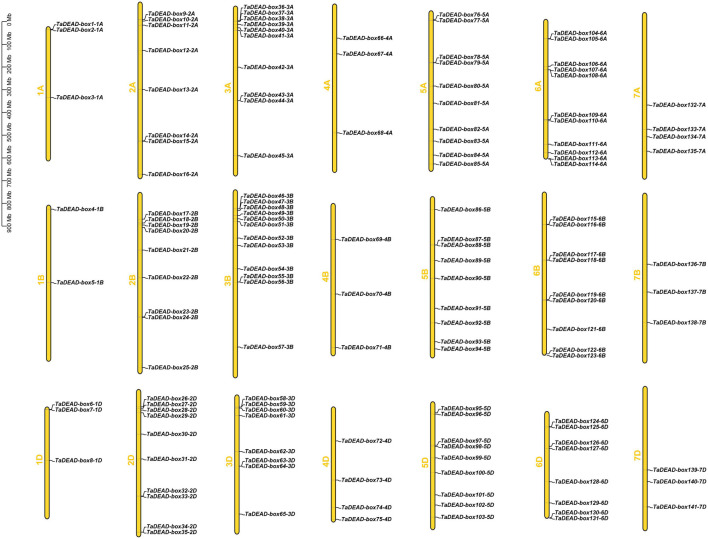
Chromosomal distribution of the 141 *TaDEAD-box* genes. The 141 *TaDEAD-box* genes were unevenly distributed on all 21 wheat chromosomes. The chromosome numbers are denoted left of each chromosome, and the bar locates on the left side shows the size of chromosome in megabases (MB).

The 141 *TaDEAD-box* genes were unevenly distributed across all 21 wheat chromosomes (Figure 2). A total of 9, 27, 30, 10, 28, 28, and 10 genes were present on chromosomes 1 through 7, respectively, and the numbers of *TaDEAD-box* genes per chromosome ranged from 2 to 12 (Supplementary Figure 1). Chromosome 1B contained the fewest *TaDEAD-box* genes (∼1.4%), whereas chromosome 2B contained the most (∼8.5%), followed by 6A (∼7.8%). These results suggested that duplication of *TaDEAD-box* genes may have occurred more often on wheat chromosomes 2, 3, 5, and 6. *TaDEAD-box* genes clustered together in one region of the chromosome may belong to the same group. For example, *TaDEAD-box107-6A*/*TaDEAD-box108-6A* and *TaDEAD-box117-6B*/*TaDEAD-box118-6B*, which belonged to Group II, were tightly linked on chromosome 6. *TaDEAD-box55-3B*/*TaDEAD-box56-3B* and *TaDEAD-box63-3D*/*TaDEAD-box64-3D* belonged to group IX and were tightly linked on chromosome 3 (Figure 2). Chromosome translocation and inversion analysis showed that one triad (*TaDEAD-box70-4B*/*TaDEAD-box73-4D*/T*aDEAD-box66-4A*) was involved in inversion between the long and short arms of chromosome 4A, and another triad (*TaDEAD-box85-5A*/*TaDEAD-box71-4B*/*TaDEAD-box75-4D*) was involved in translocation between the long arms of chromosomes 4A and 5A (Supplementary Table 2).

**FIGURE 3:**
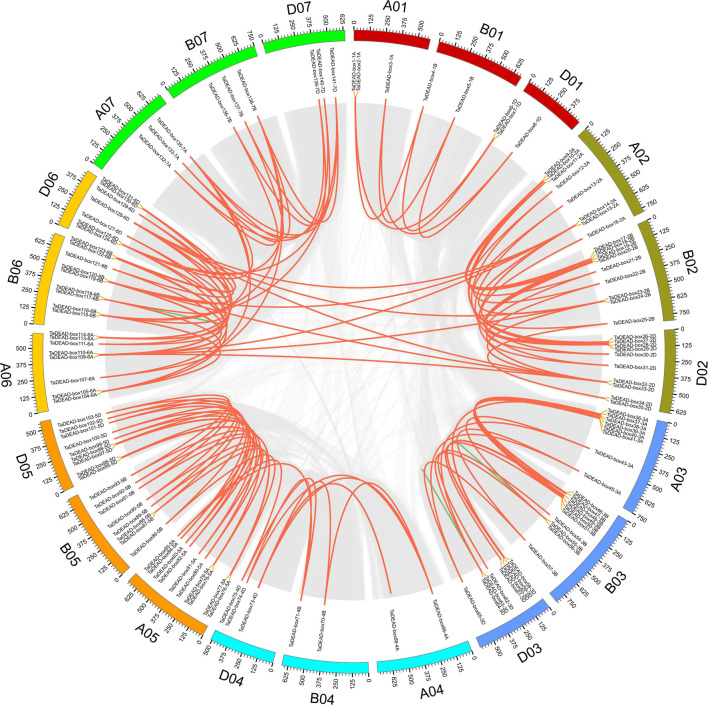
Distribution and duplication events of *TaDEAD-box* genes across wheat genome. All *TaDEAD-box* genes were mapped to 21 wheat chromosomes in a circle using Circos, and WGD/segmental and tandem duplications were mapped to their respective locations. Gray regions indicate all synteny blocks within the wheat genome, red and green lines, respectively, represent WGD/segmental and tandem duplications. The chromosome numbers are marked inside of the circle.

To elucidate the expansion mechanism within this gene family, we conducted collinearity analysis of *TaDEAD-box* genes within the wheat genome. Using the definitions of Wang M. et al. (2016) and Fan et al. (2019), we evaluated tandem and segmental duplication events of *TaDEAD-box* genes. There were 128 genes located within syntenic blocks, forming 138 pairs of duplicated genes (Figure 3 and Supplementary Table 3). Approximately 79.3% (107/135) of the duplicated *TaDEAD-box* genes were clustered on chromosomes 2, 3, 5, and 6, and Group I contained the most duplicated genes, consistent with the results above. Specifically, ∼4.3% (6/141) of *TaDEAD-box* genes that were located adjacent to one another on the same chromosome were derived from tandem duplication events, forming three tandem duplicated pairs: *TaDEAD-box55-3B*/*TaDEAD-box56-3B*, *TaDEAD-box63-3D*/*TaDEAD-box64-3D*, and *TaDEAD-box117-6B*/*TaDEAD-box118-6B*. In addition, we further observed that ∼88.7% (125/141) of *TaDEAD-box* genes were derived from whole genome duplication (WGD)/segmental duplication, such as *TaDEAD-box135-7A*/*TaDEAD-box138-7*. It thus appears that WGD/segmental duplication is the main driving factor behind expansion of *TaDEAD-box* genes in the wheat genome.

**FIGURE 4:**
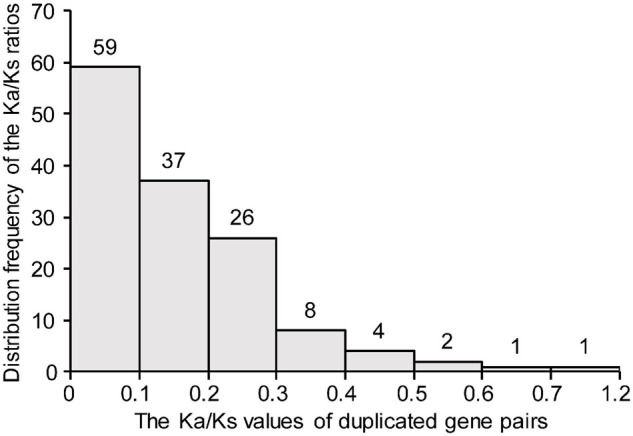
Histogram of distribution frequency of pairwise Ka/Ks ratios for duplicated *TaDEAD-box* genes. Ka, nonsynonymous substitution rate; Ks, synonymous substitution rate.

To investigate the evolutionary forces acting on the *TaDEAD-box* genes, the Ka/Ks ratios were calculated for duplicate gene pairs. The Ka/Ks ratios of all except one duplicate pair were less than 1 and ranged from 0 (*TaDEAD-box135-7A*/*TaDEAD-box138-7B*) to 0.62 (*TaDEAD-box117-6B*/*TaDEAD-box127-6D*) (Figure 4). The Ka/Ks ratios of the three *TaDEAD-box* tandem duplicated gene pairs ranged between 0.22 and 0.54 (Supplementary Table 3). These results revealed that most duplicated *TaDEAD-box* genes were under purifying selection.

### Gene Structure and Conserved Motif Analysis of TaDEAD-box Members

**FIGURE 5:**
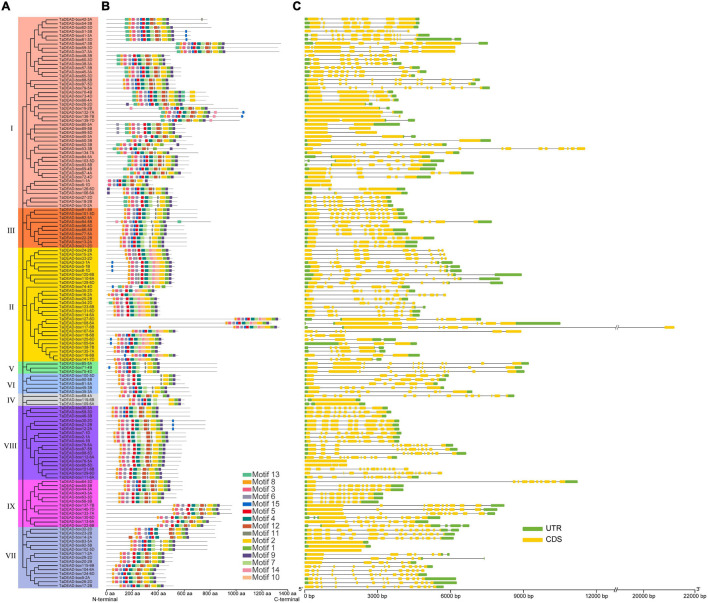
Phylogenetic tree, motif distribution and gene structure analysis of 141 TaDEAD-box members. **(A)** Phylogenetic tree of DEAD-box members in wheat. A total of 141 TaDEAD-box proteins were used to construct the neighbor-joining (NJ) phylogenetic tree by MEGA X with 1,000 bootstrap replicates. The different groups are marked with different colors. **(B)** Motif distribution of 141 TaDEAD-box proteins. The different colored boxes represent 15 motifs, and the box lengths represent motif lengths. **(C)** The exon-intron structure analysis of 141 *TaDEAD-box* genes. Exon and introns lengths are displayed proportionally, yellow boxes represent exons, black lines represent introns, and green boxes represent 5′ and 3′ UTR.

Exon-intron structures and conserved motifs were compared between the 141 TaDEAD-box members (Figure 5). Differences in exon-intron structure are associated with the evolution and function among members of the gene family. Except for five genes that contained no intron, *TaDEAD-box* genes had between 1 and 15 introns (Figure 5 and Supplementary Table 4). *TaDEAD-box32-2D* harbored the most exons and introns (16 and 15, respectively). Members of Groups I and II had the most introns, followed by Group VIII and VII. Members of Group I ranged from 1-13 and had an average of six introns, except for one gene with no intron. Members of Group II had between three and nine introns, with 75% (21/28) having six or more. There was significant variation among members of Group VII; three genes had no intron, three genes contained five to six introns, and the others contained more than 11. In Group VIII, almost 88.9% (16/18) genes contained 7 or more introns, except for one gene contained two introns and the other with no intron (Figure 5). Genes in the same branch were homeologs and had similar gene structure, suggesting that exon-intron structure was highly correlated with phylogenetic relationship. However, a few exceptions existed among homologous genes; for example, *TaDEAD-box130-6D*, *TaDEAD-box113-6A*, and *TaDEAD-box122-6B* had differences in exon and intron numbers.

Fifteen conserved motifs with 6-50 amino acids each were identified using MEME (Figure 5 and Supplementary Table 5). Motifs 1, 2, 3, 4, 5, 6, 8, 7, and 9 were present in almost all proteins, and motif 4 contained the highly conserved sequence residues Asp-Glu-Ala-Asp (D-E-A-D) (Figure 5). In addition, motif 11 was present in all members, with the exception of Group II, where less than 22% members contained motif 11. However, some motifs were specific to individual groups; for example, motif 10 was only found in Group II, and motifs 13 and 14 were only found in Groups I, II, and III. Group-specific motifs such as these may account for functional diversity of individual groups. Interestingly, the identified motifs were concentrated close to the C-terminal in TaDEAD-box108-6A/TaDEAD-box117-6B/TaDEAD-box 127-6D (Group II) and TaDEAD-box133-7A/TaDEAD-box137-7B/TaDEAD-box140-7D (Group IX). These results indicate that TaDEAD-box proteins in the same subclade often had similar motif composition, which was consistent with their phylogenetic relationship. However, a few exceptions were found in homologous proteins. For example, motif 3 was found in TaDEAD-box115-6B and TaDEAD-box104-6A, but not in TaDEAD-box124-6D. Within the same branch, the proteins contained different motifs, and the genes encoding them also differed in the arrangement of introns and exons (e.g., *TaDEAD-box1-1A*/*TaDEAD-box6-1D*, *TaDEAD-box56-3B*/*TaDEAD-box63-3D*, and *TaDEAD-box136-7B*/*TaDEAD-box139-7D*. These differences may be due to the evolution of the *TaDEAD-box* members in wheat. Overall, our phylogenetic analysis revealed that phylogeny and function were associated with the diversity of exon-intron structure and structural motif distribution.

### Promoter *Cis*-Element Analysis of *TaDEAD-box* Genes

Gene expression is often regulated by *cis*-elements in the promoter region. We found 3,178 potential *cis*-elements in the promoter regions of the 141 *TaDEAD-box* genes (Supplementary Table 6). These were divided into four broad categories: light-responsive elements (44.0%), hormone-responsive elements (40.1%), environmental stress-responsive elements (8.5%), and plant growth-related elements (7.4%) (Supplementary Figure 2). Among the 269 environmental stress-responsive elements, the proportion of low temperature-responsive elements was highest (45.4%), followed by drought response (40.9%), and wound-responsive elements accounted for the lowest proportion (1.1%). Among the 1275 hormone-responsive elements, methyl jasmonate (MeJA)-responsive elements accounted for the highest proportion (46.0%), with a lower proportion of abscisic acid-responsive elements (33.0%). These results show that TaDEAD-box genes may be involved in plant abiotic stress responses, especially low temperature and drought stress (Supplementary Figure 2). In addition, among the plant growth-related elements, meristem expression elements were the most abundant, followed by metabolism regulation elements, suggesting involvement of *TaDEAD-box* genes in plant growth and development.

### Expression Patterns Analysis of *TaDEAD-box* Genes

**FIGURE 6:**
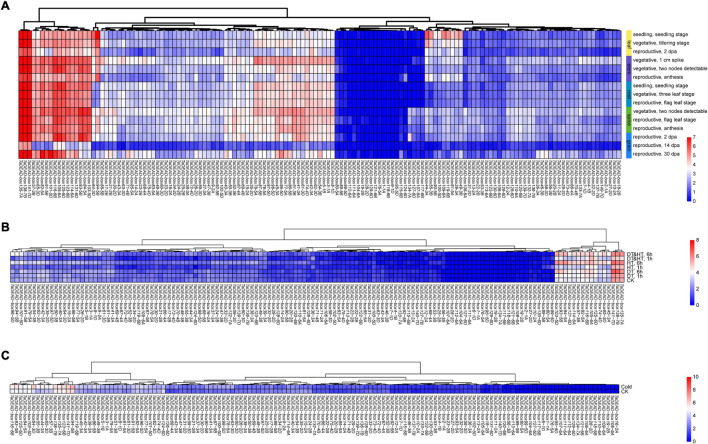
Hierarchical clustering of expression patterns of 141 *TaDEAD-box* genes using transcriptome data. The expression pattern heatmaps of *TaDEAD-box* genes were generated through pheatmap package of R project13. The expression levels are indicated by the different color bars, red: higher expression, blue: lower expression. **(A)** Expression patterns of 141 *TaDEAD-box* genes in different tissues and development stages. Columns represent 141 *TaDEAD-box* genes, and rows represent different developmental tissues/stages, including leaf, stem, root, spike, and grain. **(B)** Expression patterns of 141 *TaDEAD-box* genes under drought and high-temperature treatments. Columns represent 141 *TaDEAD-box* genes, and rows represent control and stress treatments. **(C)** Expression patterns of 141 *TaDEAD-box* genes under cold treatment. Columns represent 141 *TaDEAD-box* genes, and rows represent control and cold treatment. CK, control check; DT, drought treatment; HT, high-temperature treatment.

In order to study the expression patterns of *TaDEAD-box* genes in different tissues and developmental stages, transcriptomic data (choulet_URGI) were analyzed and a tissue-specific expression clustering heatmap generated (Figure 6A and Supplementary Table 7). Some genes displayed low transcription levels, including *TaDEAD-box80-5A*, *89-5B*, *99-5D*, *112-6A*, *111-6A*, *104-6A*, and *19-2B*, which clustered together, and *TaDEAD-box6-1D*, which was not expressed in the examined tissues and developmental stages. Some genes exhibited tissue-specific expression, for instance, *TaDEAD-box44-3A*, *55-3B*, *90-5B*, *100-5D*, *109-6B*, *81-5A*, *39-3A* and *49-3B*, which clustered together and were highly expressed in the leaf. However, some genes were constitutively expressed in wheat: *TaDEAD-box135-7A*, *138-7B*, *141-7D*, *57-3B*, *65-3D*, *82-5A*, *91-5B*, *101-5D*, *116-6B*, *105-6A*, *125-6D*, *123-6B*, *114-6A*, *131-6D*, *93-5B*, *84-5A*, and *103-5D* clustered together and displayed high transcription levels in most tissues throughout plant development (Figure 6A). In general, *TaDEAD-box* genes exhibited a great deal of tissue-specific expression, indicating that they may be involved in adaptation to different physiological processes.

In order to analyze the expression patterns of *TaDEAD-box* genes under abiotic stresses, transcriptomic data (SRP045409, SRP043554) were analyzed with an abiotic stress expression clustering heatmap (Figures 6B,C and Supplementary Table 7). A large number of genes were expressed at low levels under drought, high temperature, and cold treatments, including *TaDEAD-box117-6B*, *127-6D*, *118-6B*, and *19-2B*, which clustered together. *TaDEAD-box1-1A*, *6-1D*, *76-5A*, and *95-5D* were not detectable at all either before or after stress treatments. However, 16 genes were differentially expressed under drought and high-temperature treatments (*TaDEAD-box135-7A*, *138-7B*, *141-7D*, *45-3A*, *65-3D*, *74-4D*, *125-6D*, *105-6A*, *116-6B*, *82-5A*, *57-3B*,*114-6A*, *131-6D*, *84-5A*, *93-5B*, *103-5D*) (Figure 6B), and a further 15 genes were differentially expressed under cold treatment (*TaDEAD-box94-5B*, *141-7D*, *123-6B*, *135-7A*, *138-7B*, *57-3B*, *65-3D*, *44-3A*, *55-3B*, *64-3D*, *103-5D*, *84-5A*, *125-6D*, *93-5B*, *116-6B*) (Figure 6C). We selected 10 genes for further analysis that responded to abiotic stresses and were highly expressed throughout growth and development: *TaDEAD-box141-7D*, *135-7A*, *138-7B*, *57-3B*, *65-3D*, *125-6D*, *103-5D*, *93-5B*, *84-5A*, and *116-6B*.

### *TaDEAD-box* Genes Were Involved in Abiotic Stress Responses

**FIGURE 7:**
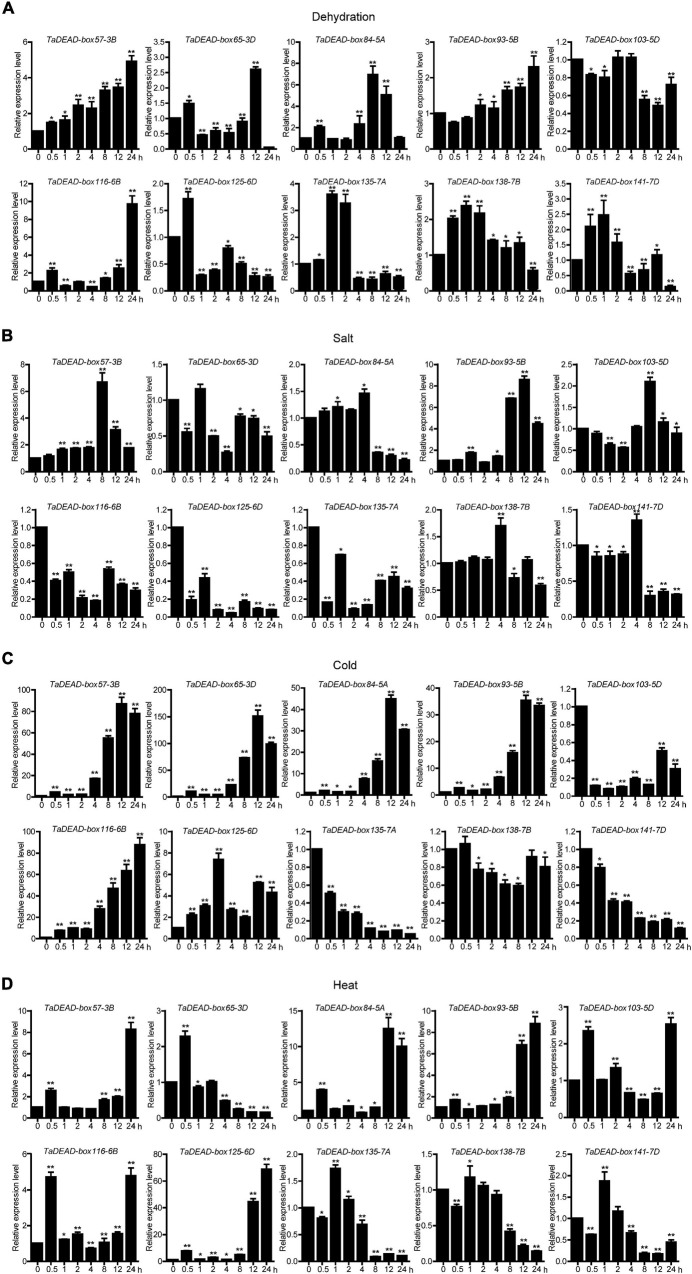
Quantitative Real-Time PCR (qRT-PCR) analysis of 10 *TaDEAD*-box genes under dehydration **(A)**, salt **(B)**, cold **(C)** and heat **(D)** treatments. Seven-day-old wheat seedlings were used for dehydration (250 mM Mannitol), salt (200 mM NaCl), heat (42°C), and cold (4°C) stress treatments. The wheat β*-actin* was used as an internal control. The data represent means ± SD of three biological replications. ANOVA test was used to analyze significant differences (*p < 0.05, **p < 0.01).

Expression of the 10 selected genes was studied at a finer scale with qRT-PCR (Figure 7), and the primers are listed in Supplementary Table 8. Results showed differing responses to abiotic stresses. Under dehydration treatment, *TaDEAD-box57-3B*, *TaDEAD-box84-5A* and *TaDEAD-box116-6B* were significantly up-regulated (> 5-fold) and peaked at 24 h, 8 h, and 24 h, respectively (Figure 7A). Aside from *TaDEAD-box103-5D*, the remaining six genes showed a slight response to dehydration treatment (Figure 7A). After salt treatment, *TaDEAD-box57-3B* and *TaDEAD-box93-5B* were up-regulated (> 5-fold), whereas *TaDEAD-box116-6B*, *TaDEAD-box125-6D*, and *TaDEAD-box135-7A* were down-regulated (Figure 7B). Under cold stress, five genes were significantly up-regulated (> 30-fold); remarkably, *TaDEAD-box57-3B*, *TaDEAD-box65-3D*, and *TaDEAD-box116-6B* were up-regulated more than 80-fold, suggesting that these genes may play an important role in cold response (Figure 7C). However, *TaDEAD-box135-7A*, *TaDEAD-box103-5D*, and *TaDEAD-box141-7D* were down-regulated after cold treatment (Figure 7C). Following heat treatment, five genes were up-regulated (> 5-fold); *TaDEAD-box125-6D* showed the highest expression levels (> 60-fold) after heat treatment for 24 h (Figure 7D). *TaDEAD-box57-3B* was significantly up-regulated under all tested treatments and was therefore selected for further analysis.

### TaDEAD-box57-3B Was Located in Cytoplasm and Plasma Membrane

**FIGURE 8:**
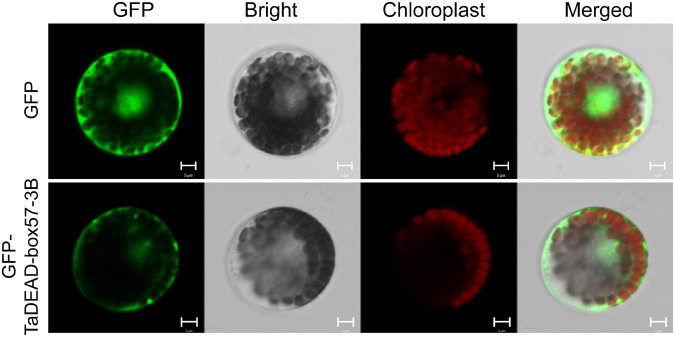
Subcellular localization of TaDEAD-box57-3B. The p16318hGFP control vector and recombinant constructs were transiently expressed in wheat protoplasts. The green indicates GFP signals, and the red indicates chloroplast autofluorescence. Results were observed after transformation for 18 h with confocal microscopy. Scale bars = 5 μm.

Subcellular localizations of the 141 TaDEAD-box family members were predicted through using BUSCA (Supplementary Table 1). For transient expression assays, the p16318hGFP control and recombinant plasmids were transformed into wheat protoplasts mediated by PEG4000. GFP expression in protoplasts was observed using a confocal laser scanning microscope. Relative to the control where GFP expression was dispersed throughout the cell, TaDEAD-box57-3B was localized in the cytoplasm and plasma membrane (Figure 8).

### Ectopic Expression of *TaDEAD-box57-3B* Enhanced Drought Tolerance in *Arabidopsis*

**FIGURE 9:**
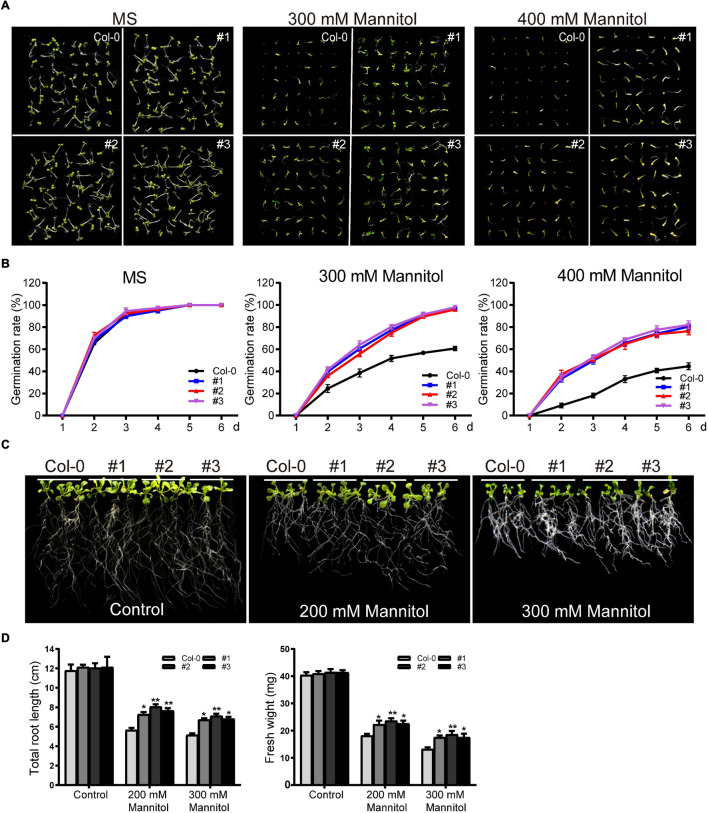
Phenotype analysis of *TaDEAD-box57-3B* transgenic plants under dehydration treatment. **(A)** Seed germination assays of WT and *TaDEAD-box57-3B* transgenic lines. Seeds were grown on MS and MS medium supplemented with 300 mM and 400 mM mannitol. **(B)** The germination rates of WT and *TaDEAD-box57-3B* transgenic lines. The germination rate was calculated for the next 6 days. **(C)** Root length assays of WT and *TaDEAD-box57-3B* transgenic lines. Five-day-old seedlings were transferred to MS medium with or without 200 mM, 300 mM mannitol. **(D)** The total root lengths and fresh weights of WT and *TaDEAD-box57-3B* transgenic lines. All the data represent the means ± SDs of three independent biological replicates and asterisks (*or **) represent the significant differences at *p* < 0.05 or *p* < 0.01(ANOVA test).

*TaDEAD-box57-3B* under the control of CaMV35S was transformed into *Arabidopsis*, and three homozygous T_3_ lines were selected for phenotypic analysis. First, transgenic and wild-type (WT) seeds were used for germination assays. After surface sterilization, transgenic and WT seeds were grown on MS medium as a control or on MS supplemented with mannitol (300 mM or 400 mM). In the control medium, there was no significant difference in germination rate between WT and transgenic *Arabidopsis*. Germination of both WT and transgenic lines was inhibited in the 300 mM mannitol treatment, although the transgenic lines showed higher germination rates than the WT (Figures 9A,B). Germination rates of WT and transgenic lines were also inhibited to varying degrees in the 400 mM mannitol treatment; for example, the germination rates of the transgenic lines on the sixth day were approximately 80%, significantly higher than the rate of the WT (∼45%) (Figures 9A,B).

To explore the function of *TaDEAD-box57-3B* on seedlings, root growth assays and drought resistance tests in soil were performed. For root growth assays, 5-day-old seedlings were transferred to MS medium containing 200 mM or 300 mM mannitol, and the total root length and fresh weight were calculated. There was no significant difference in root length or fresh weight between WT and transgenic lines in the control medium. In the 200 mM mannitol treatment, growth was inhibited for both WT and transgenic plants; however, transgenic lines showed less growth inhibition than the WT, with longer roots and higher fresh weights (Figures 9C,D). In 300 mM mannitol, growth was more severely inhibited for WT and transgenic lines; however, consistent with the results of 200 mM mannitol treatment, the effect was decreased in the transgenic plants compared to the WT for both total root lengths and fresh weights (Figures 9C,D).

**FIGURE 10:**
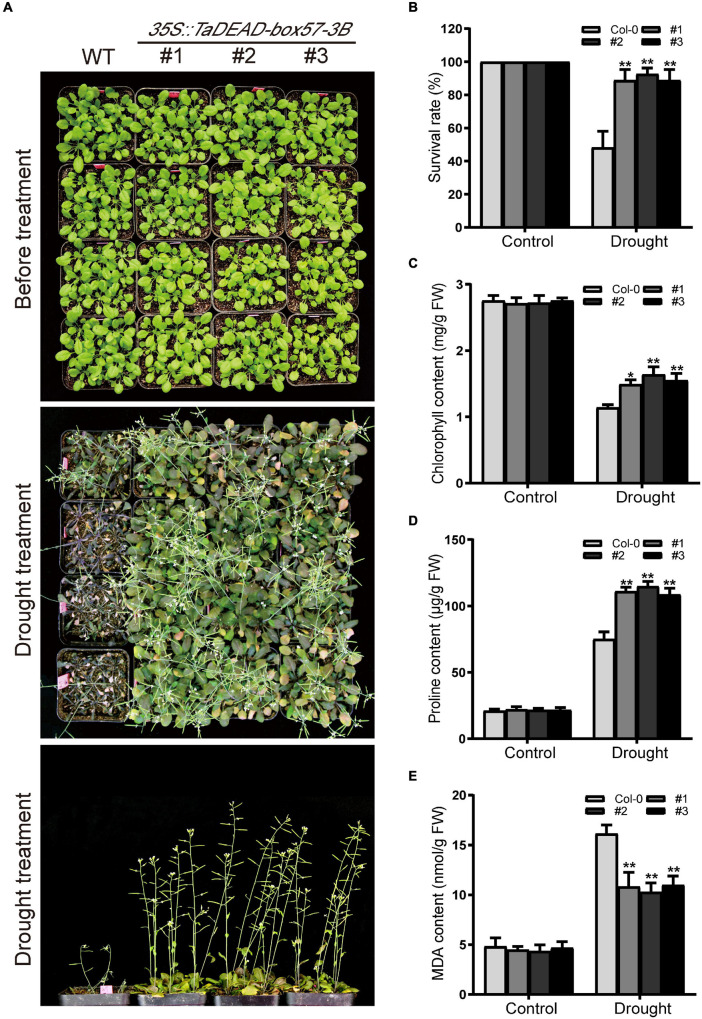
Ectopic expression of *TaDEAD-box57-3B* enhanced drought tolerance in transgenic *Arabidopsis*. **(A)** Phenotypes of 21-day-old WT and *TaDEAD-box57-3B* transgenic plants under drought stress. **(B)** The survival rates of WT and transgenic plants under drought condition was monitored 7 days after rewatering. **(C-E)** The physiological indicators of WT and transgenic plants under normal and stress treatments, including chlorophyll **(C)**, proline **(D)**, and MDA **(E)** contents. All the data represent the means ± SDs of three independent biological replicates and asterisks (*or **) represent the significant differences at *p* < 0.05 or *p* < 0.01(ANOVA test).

To measure drought resistance in soil, 21-day-old seedlings were exposed to drought conditions by withdrawing irrigation until there was a significant difference between WT and transgenic plants, after which the survival rates were counted. Before drought treatment, there was no significant difference in morphology between WT and transgenic lines. After drought treatment, the transgenic plants exhibited significantly increased drought tolerance, whereas WT plants were severely wilted and comparatively sensitive to drought treatment, with ∼48.1% survival (Figures 10A,B).

To explain the mechanism of enhanced drought tolerance, stress-related physiological indicators including levels of chlorophyll, proline, and MDA were measured under control and drought conditions. After drought treatment, levels of proline and MDA were increased and chlorophyll contents were decreased in the WT, whereas the transgenic lines showed significantly lower MDA contents but higher proline and chlorophyll content. There were no significant differences between the lines under standard conditions (Figures 10C–E). Collectively, these findings indicate that ectopic expression of *TaDEAD-box57-3B* enhanced drought tolerance in transgenic *Arabidopsis*.

### Ectopic Expression of *TaDEAD-box57-3B* Increased Salt Resistance in *Arabidopsis*

**FIGURE 11:**
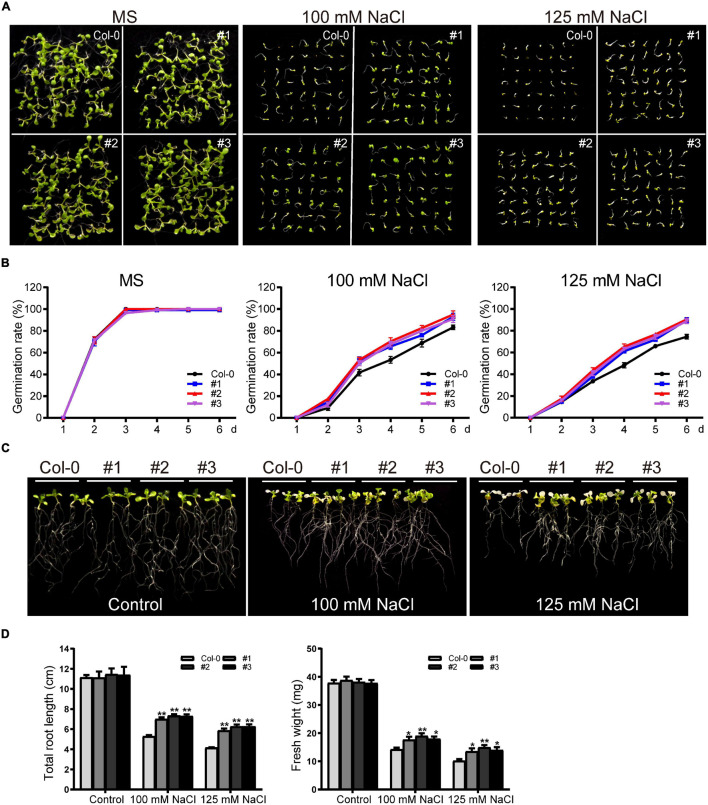
Phenotype analysis of *TaDEAD-box57-3B* transgenic plants under salt treatment. **(A)** Seed germination assays of WT and *TaDEAD-box57-3B* transgenic lines. Seeds were grown on MS and MS medium supplemented with 100 mM and 125 mM NaCl. **(B)** The germination rates of WT and *TaDEAD-box57-3B* transgenic lines. The germination rate was calculated for the next 6 days. **(C)** Root length assays of WT and *TaDEAD-box57-3B* transgenic lines. Five-day-old seedlings were transferred to MS and MS medium supplemented with 100 mM and 125 mM NaCl. **(D)** The total root lengths and fresh weights of WT and *TaDEAD-box57-3B* transgenic lines. The data are shown as the means ± SD obtained from three biological replicates. ANOVA test was used to analyze significant differences (*p < 0.05, **p < 0.01).

For germination assays, WT and transgenic seeds were grown on standard MS medium or MS supplemented with NaCl (100 mM or 125 mM) after surface sterilization. There was no significant difference in germination rates between WT and transgenic plants in the control group. In the salt-treated plants, germination was inhibited in both the WT and transgenic lines; consistent with our drought treatment results, the transgenic lines showed higher germination rates (Figures 11A,B). Germination was inhibited to varying degrees for WT and transgenic plants grown in 125 mM NaCl, with the germination rates of transgenic lines at six days ∼89.7%, significantly higher than the rate of the WT (∼74.5%) (Figures 11A,B).

We next performed root growth assays and drought resistance tests in soil to identify the role of *TaDEAD-box57-3B* in salt stress response. After 5-day-old seedlings were transferred to MS medium supplemented with 100 mM and 125 mM NaCl for another 7 days, we measured the total root lengths and fresh weights. No significant difference was observed between WT and transgenic lines under normal conditions. However, in those treated with 100 mM NaCl, growth was inhibited in both WT and transgenic plants. Again, the transgenic lines showed significantly better growth than WT, with longer roots and higher fresh weights (Figures 11C,D). Treatment with 125 mM NaCl caused poor growth in the WT and transgenic lines; plants were bleached and had shorter roots and lower fresh weights than those grown in the control medium, but all of these effects were less severe in the transgenic lines compared to the WT (Figures 11C,D).

**FIGURE 12:**
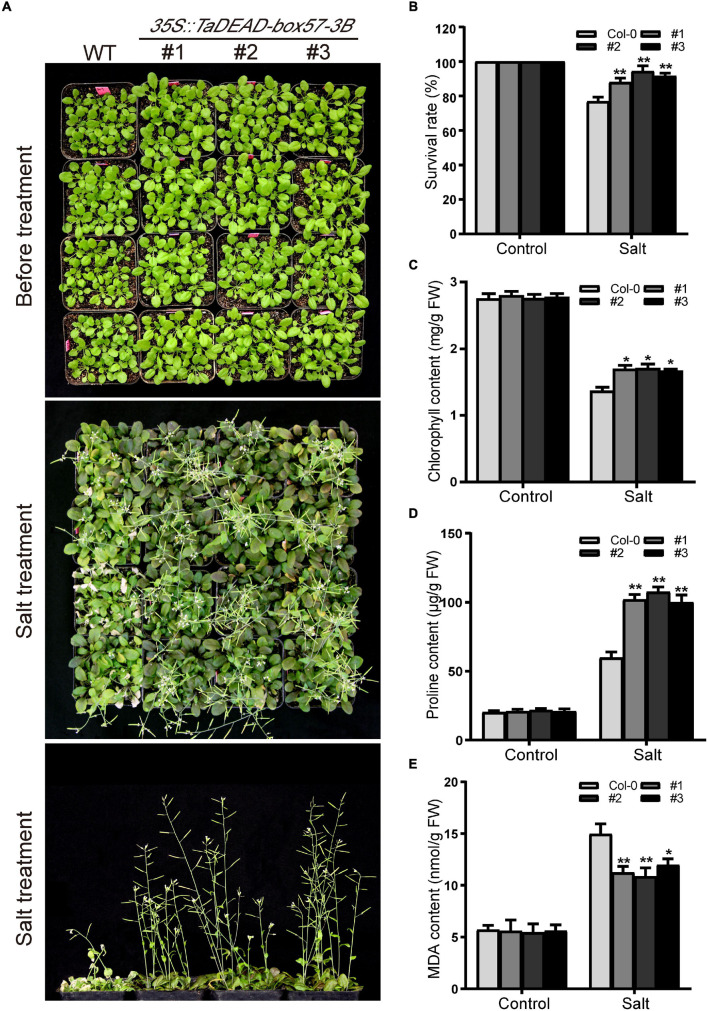
Ectopic expression of *TaDEAD-box57-3B* increased salt resistance in transgenic *Arabidopsis*. **(A)** Phenotypes of 21-day-old WT and *TaDEAD-box57-3B* transgenic plants under salt stress. **(B)** The survival rates of WT and transgenic plants under salt treatment. **(C–E)** The physiological indicators of WT and transgenic plants under normal and stress treatments, including chlorophyll **(C)**, proline **(D)**, and MDA **(E)** contents. The data are shown as the means ± SD obtained from three biological replicates. ANOVA test was used to analyze significant differences (*p < 0.05, **p < 0.01).

In order to determine the level of salt resistance in soil, 21-day-old plants were irrigated with 200 mM NaCl solution and the survival rates were counted. There was no obvious difference in morphology between the WT and transgenic lines before salt treatment (Figure 12A), and after treatment, transgenic plants exhibited increased salt resistance compared to the WT (Figure 12B). Later, the physiological indicators (chlorophyll, proline, and MDA contents) were measured under normal and salt treatment. In the control treatment, there was no obvious difference in physiological indicators. After salt treatment, the chlorophyll contents were decreased in both WT and transgenic plants, but transgenic lines looked greener with higher chlorophyll contents than the WT (Figure 12C). Proline and MDA contents were increased in all genotypes under salt treatment, but the transgenic lines showed significantly lower MDA contents and higher proline levels compared to the WT (Figures 12D,E). These results demonstrated that ectopic expression of *TaDEAD-box57-3B* in *Arabidopsis* contributed to a stronger capacity to resist salt stress.

### Ectopic Expression of *TaDEAD-box57-3B* Increased Cold Tolerance in *Arabidopsis*

**FIGURE 13:**
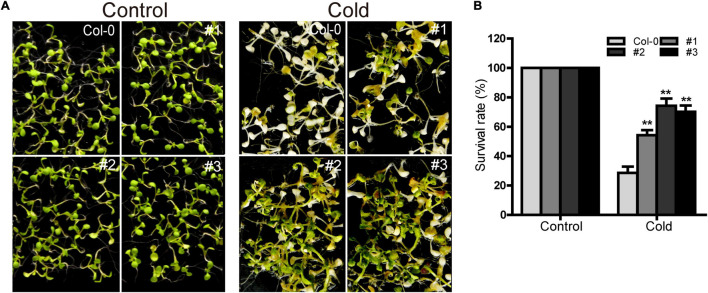
Ectopic expression of *TaDEAD-box57-3B* increased cold tolerance in transgenic *Arabidopsis*. **(A)** Phenotypes of WT and *TaDEAD-box57-3B* transgenic plants under cold stress. **(B)** Survival rates of WT and *TaDEAD-box57-3B* transgenic lines under cold stress. Five-day-old seedlings were placed at 4°C for 1 d, –8°C for 1 h, 4°C for 12 h and then resumed at 22°C. The data are shown as the means ± SD obtained from three biological replicates. ANOVA test was used to analyze significant differences (**p < 0.01).

*TaDEAD-box57-3B* was up-regulated more than 80-fold in cold treatment compared to standard growth conditions, suggesting that it may play an important role in cold response. Using the three *TaDEAD-box57-3B* overexpressing lines, we observed the phenotypes of the WT and transgenic lines under cold stress. There was no significant difference between WT and transgenic plants under normal conditions (Figure 13). However, in response to cold treatment, the transgenic lines showed significantly improved growth compared to the WT in terms of survival rates (> 50% vs. < 30%, respectively). These results suggest that ectopic expression of *TaDEAD-box57-3B* may improve cold tolerance in transgenic *Arabidopsis*.

## Discussion

DEAD-box RNA helicases play an important role in RNA metabolism (Bowers et al., 2006; Cordin et al., 2006; Linder, 2006; Linder and Fuller-Pace, 2013; Putnam and Jankowsky, 2013), and are widely involved in plant growth, development, and stress responses (Guan et al., 2013; Khan et al., 2014; Bush et al., 2016; Liu et al., 2016; Paieri et al., 2018; Chen D. et al., 2020; Lu et al., 2020). However, knowledge of DEAD-box RNA helicases has so far been limited in wheat. Here, we conducted a comprehensive analysis of the *DEAD-box* gene family in wheat, and characterized the function of an abiotic stress-responsive family member, *TaDEAD-box57-3B*. This work provides a foundation for better understanding the roles of TaDEAD-box members in plant growth, development, and stress responses.

TaDEAD-box family members are more numerous in wheat than in *Arabidopsis*, rice, tomato, and maize by nearly 2.47-fold, 3-fold, 3.36-fold, and 2.47-fold, respectively. This may be due to a higher homoeolog retention rate in wheat. DEAD-box genes are unevenly distributed across the chromosomes; for example, chromosome 1 contains the most DEAD-box genes in *Arabidopsis* and rice, and only one DEAD-box gene was located in particular chromosome among these species (Xu et al., 2013a,b; Wan et al., 2020). In wheat, the 141 *TaDEAD-box* genes were also unevenly distributed on all 21 chromosomes (Figure 2). Whole genome duplication (WGD) is a large-scale process of gene multiplication at the chromosomal level that can generate abundant duplicated genes, playing a vital role in the expansion of some gene families (Schmutz et al., 2010; Lee et al., 2013; Su et al., 2020; Xu et al., 2021). For example, the significant expansion of *TaSCPL* genes in the wheat genome is attributed to tandem duplication (10.5%) and WGD/segmental duplication (64.8%). Duplicate *MADS-box* genes may be derived from tandem or segmental duplication, which would produce many homologs on different chromosomes (Duan et al., 2015; Schilling et al., 2020; Zhao et al., 2021). Expansion of *ANK* genes in the soybean genome is also attributed to tandem and segmental duplication events (35.8% and 46.0%, respectively) (Zhao et al., 2020). Consistent with these prior findings, ∼4.3% and 88.7% of *TaDEAD-box* genes were determined to be derived from tandem and WGD/segmental duplication events, respectively. This suggests that WGD/segmental duplication is the main driving factor for expansion of *DEAD-box* genes in the wheat genome (Figure 3). Gene family expansion is hypothesized to contribute to the ability of a species to adapt to various environments, thus enabling their wide distribution (Theissen et al., 2016; Zhao et al., 2021).

Overall, the diversity of gene structure and structural motifs was strongly associated with the evolutionary relationships and functions among members of the gene family. Genes in the same branch of the phylogenetic tree constructed from *DEAD-box* genes were generally homoeologs with similar gene structure; however, there were a few exceptions (Figure 5). In fact, structural divergences commonly exist in gene families and can generate homologous genes with different functions, although the mechanism is still unclear (Xu et al., 2012; Zhao et al., 2021). Nine motifs were present in almost all of the TaDEAD-box proteins, with motif 4 containing the highly conserved D-E-A-D sequence. Some motifs were restricted to specific groups within the gene family, e.g., motif 10 was specific to Group II. As expected, proteins in the same branch of the phylogenetic tree generally exhibited similar motif distribution (Figure 5). However, some proteins within the same branch contained different motifs, which may be due to differences in the arrangement of introns and exons.

*Cis*-elements in the promoter region are highly related to the regulation of gene expression. There were several stress response-related *cis*-elements in 10 *TaDEAD-box* genes identified as having different responses to abiotic stressors, indicating that these genes may be involved in plant abiotic stress responses. These results provide a foundation for further functional identification of *TaDEAD-box* genes in wheat. Increasing evidence has indicated that DEAD-box RNA helicases play important roles in plant abiotic stress responses (Khan et al., 2014; Bush et al., 2016; Huang et al., 2016a; Wang D. et al., 2016; Chen D. et al., 2020; Lu et al., 2020). Some abiotic stress-related DEAD-box RNA helicases have been characterized; for example, *RCF1* is essential for pre-mRNA splicing and cold response in plants (Guan et al., 2013). AtRH7 interacts with AtCSP3, which acts as an RNA chaperone involved in cold adaptation, affecting rRNA biogenesis and playing an important role in plant growth under cold stress (Liu et al., 2016). Rice *OsBIRH1* increases oxidative stress tolerance in transgenic plants by elevating expression levels of the oxidative defense genes *AtApx1*, *AtApx2*, and *AtFSD1* (Li et al., 2008). Rice *OsRH58* is induced by salt, drought, and heat stress, and can act as an RNA chaperone to improve salt or drought stress tolerance by increasing chloroplast mRNA translation (Nawaz and Kang, 2019). Rice OsRH42, which is located in the splicing speckles, interacts with U2 small nuclear RNA, thus playing a crucial role in accurate pre-mRNA splicing and cold adaptation in rice (Lu et al., 2020).

Transcriptomic data showed that *TaDEAD-box57-3B* was significantly up-regulated under diverse abiotic stresses, and it was therefore selected for further experimental analysis. Under drought or salt stress, *TaDEAD-box57-3B* transgenic *Arabidopsis* plants showed higher germination rates, root lengths, fresh weights and survival rates; moreover, transgenic lines had significantly better growth and higher survival rates than the WT after cold treatment. Chlorophyll content was measured as a proxy for the degree of cellular injury, and MDA content was quantified as a measure of membrane lipid peroxidation (Gunes et al., 2011; Hong et al., 2016; Wang T. T. et al., 2020; Chen et al., 2021). Proline, a common osmoprotectant, is a regulator of osmotic pressure to maintain plant homeostasis in responses to adverse conditions, and generally increases in response to abiotic stress (Szekely et al., 2008; Su et al., 2020; Wang et al., 2021; Zhao et al., 2021). These three components (chlorophyll, MDA, and proline content) can be used as physiological indicators to measure plant stress resistance. Drought and salt stress can cause oxidative and hyperosmotic damage (Zhu, 2016; Gong et al., 2020; Chen et al., 2021; Xu et al., 2021). We therefore measured these stress-related physiological indicators under normal (control) growth conditions and drought or salt treatment. Transgenic lines showed significantly lower MDA, higher proline, and higher chlorophyll contents than WT plants after drought or salt treatment, whereas there were no obvious differences in the control treatment. The higher accumulation of proline and chlorophyll and the lower MDA content may contribute to the tolerance of *TaDEAD-box57-3B* transgenic plants to drought and salt stresses. Collectively, our results indicate that *TaDEAD-box57-3B* plays a positive role in drought, salt, and cold responses. It is still unclear that whether *TaDEAD-box57-3B* affects chlorophyll, proline and MDA contents through RNA metabolism, therefore, further studies are necessary to clarify the possible role of *TaDEAD-box57-3B* in RNA metabolism under stresses.

To ensure sustainable production of wheat, crop varieties with enhanced stress tolerance should be cultivated. *TaDEAD-box57-3B* is a potential candidate gene for improving abiotic stress resistance, although further studies are required to identify the function and stress response mechanism in wheat. Discovering novel stress-responsive genes, as we have done here, and studying the mechanisms of action provides a theoretical basis and technical support for crop molecular breeding.

## Conclusion

We performed a comprehensive genome-wide analysis of the DEAD-box RNA helicase family in wheat, including analyses of phylogeny, chromosomal distribution, duplication events, gene structure, and protein motifs. A total of 141 *TaDEAD-box* genes were identified and expression patterns compared using transcriptomic data. Expression levels of 10 genes were confirmed by qRT-PCR; among them, *TaDEAD-box57-3B* was significantly up-regulated under diverse abiotic stresses, and further analysis indicated it was involved in tolerance to drought, salt, and cold treatments through regulating the degree of membrane lipid peroxidation. This study provides new insights for understanding the evolution and function of the *TaDEAD-box* gene family.

## Data Availability Statement

The datasets presented in this study can be found in online repositories. The names of the repository/repositories and accession number(s) can be found in the article/Supplementary Material.

## Author Contributions

Z-SX coordinated the project, conceived and designed the experiments, and edited the manuscript. J-NR performed the experiments and wrote the first draft. Z-HH, LZ, QZ, F-ZW, JC, Y-BZ, and MC conducted data analysis. Y-JX and Y-ZM contributed with valuable discussions. All authors reviewed and approved the final manuscript.

## Conflict of Interest

The authors declare that the research was conducted in the absence of any commercial or financial relationships that could be construed as a potential conflict of interest.

## Publisher’s Note

All claims expressed in this article are solely those of the authors and do not necessarily represent those of their affiliated organizations, or those of the publisher, the editors and the reviewers. Any product that may be evaluated in this article, or claim that may be made by its manufacturer, is not guaranteed or endorsed by the publisher.

## Footnotes

1
https://www.arabidopsis.org/

2
http://rice.uga.edu/

3
https://www.ncbi.nlm.nih.gov/

4
http://plants.ensembl.org/index.html

5
https://www.ncbi.nlm.nih.gov/Structure

6
http://pfam.xfam.org/

7
https://web.expasy.org/compute_pi/

8
http://busca.biocomp.unibo.it/

9
http://meme-suite.org/tools/meme

10
http://bioinformatics.psb.ugent.be/webtools/plantcare/html/

11
http://www.wheat-expression.com

## Supplementary Material

Supplementary Figure 1 The distribution frequency of *TaDEAD-box* genes per chromosome.

Supplementary Figure 2 *Cis*-elements distribution of 141 *TaDEAD-box* genes. **(A)** Distribution of *cis*-elements in different biological processes. **(B)** Hormone responsive *cis*-elements. **(C)** Stress related *cis*-elements. **(D)** Plant growth related *cis*-elements.

Supplementary Table 1 Detailed information of 141 *wheat DEAD-box* genes identified in wheat genome.

Supplementary Table 2 Chromosome inversion and translocation events in *TaDEAD-box* homoeologs.

Supplementary Table 3 Ka, Ks, and Ka/Ks values of duplicated *TaDEAD-box* genes.

Supplementary Table 4 Exon and intron numbers of 141 *TaDEAD-box* genes.

Supplementary Table 5 List of conserved motifs in*TaDEAD-box* proteins.

Supplementary Table 6 *Cis*-elements in the promoter region of 141 *TaDEAD-box* genes.

Supplementary Table 7 The TPM values of 141 *TaDEAD-box* genes from transcriptome data.

Supplementary Table 8 The primers used in this study.
