# Supplementary figures and images for: Genome-Wide Analysis of DEAD-box RNA Helicase Family in Wheat (Triticum aestivum) and Functional Identification of TaDEAD-box57 in Abiotic Stress Responses
Source: Front Plant Sci. 2021 Dec 9;12:797276. doi: 10.3389/fpls.2021.797276 (PMC8699334; doi:10.3389/fpls.2021.797276)

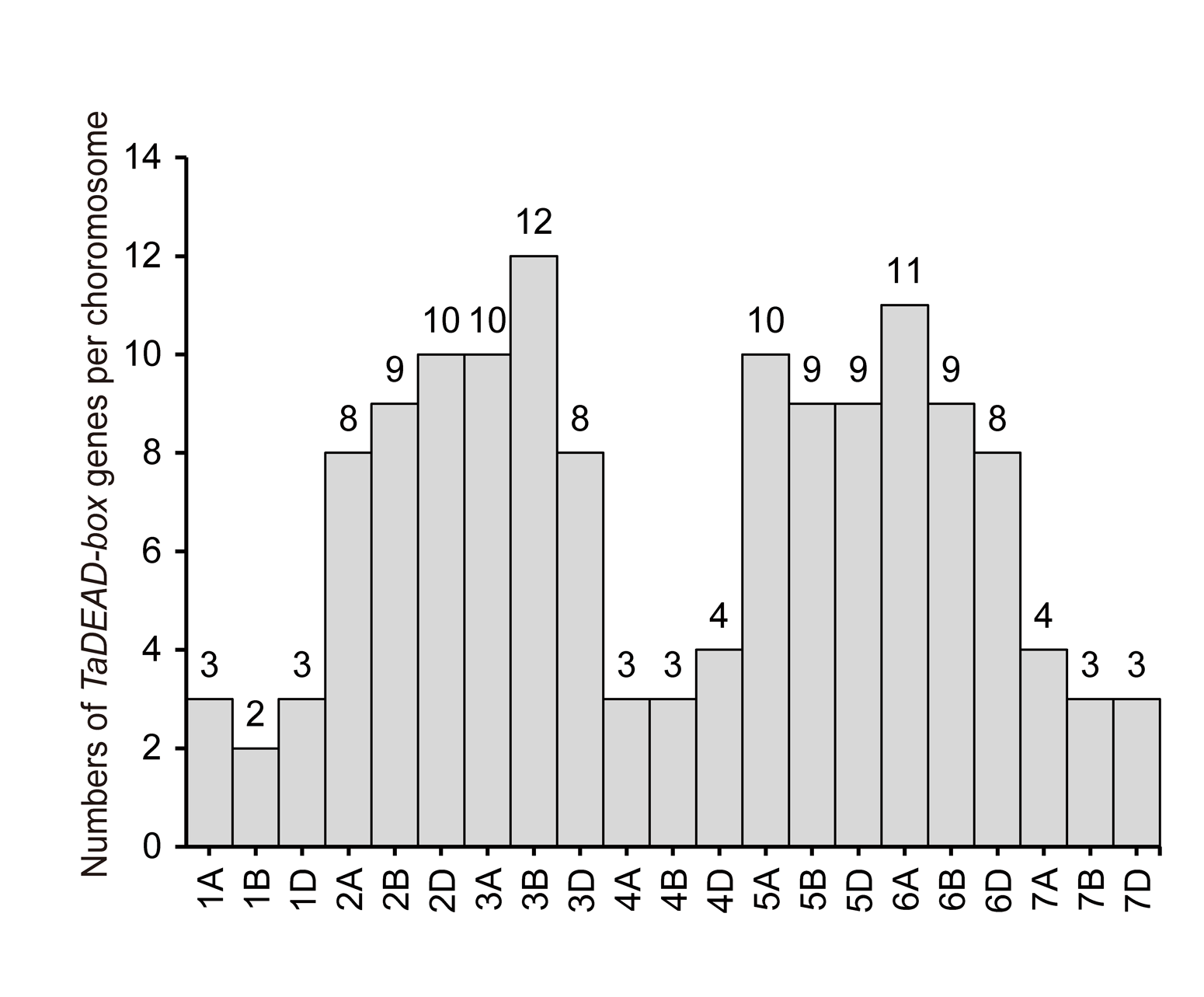

Supplement: Supplementary Figure 1 — The distribution frequency of TaDEAD-box genes per chromosome. [file Image_1.TIF]

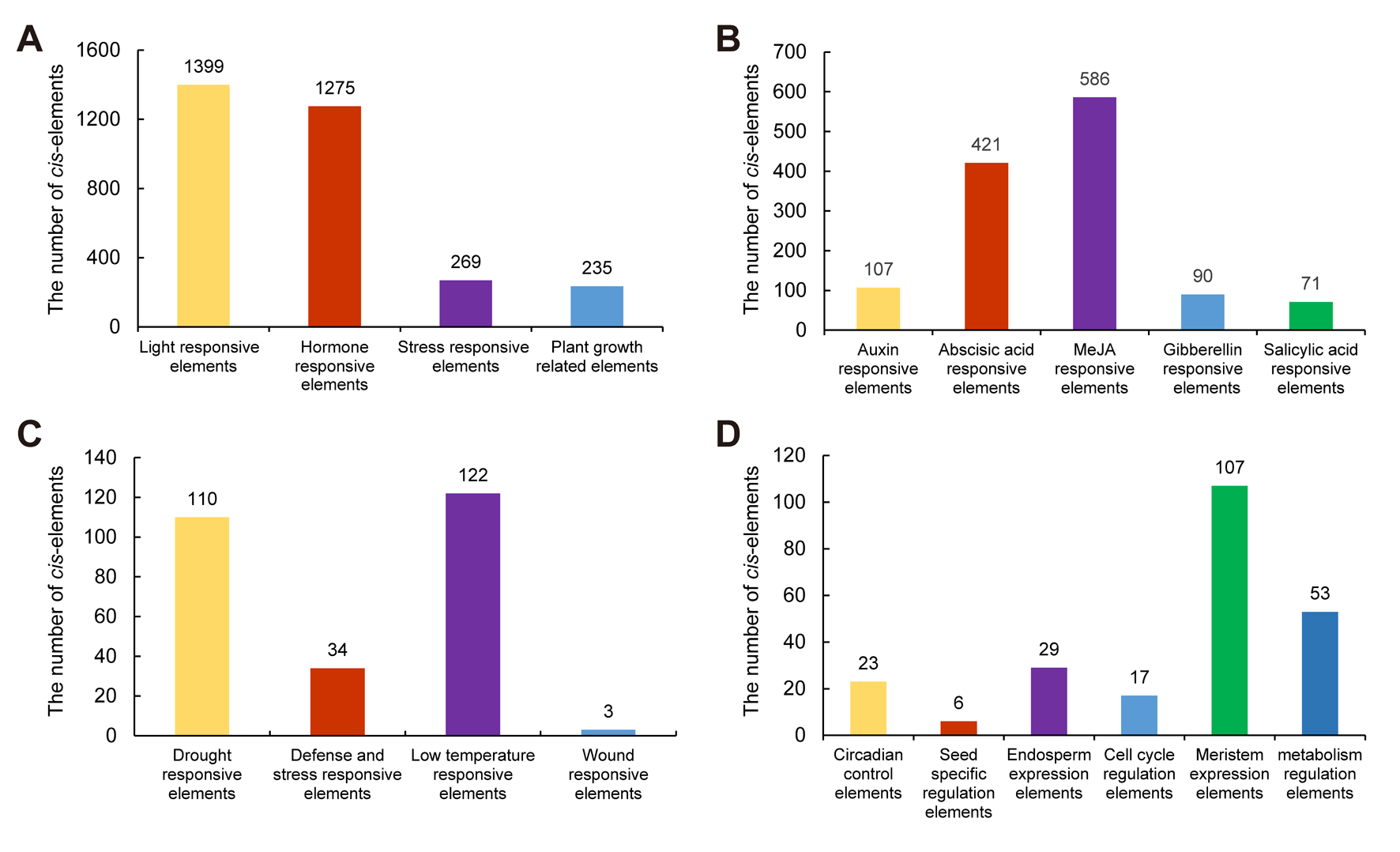

Supplement: Supplementary Figure 2 — Cis-elements distribution of 141 TaDEAD-box genes. (A) Distribution of cis-elements in different biological processes. (B) Hormone responsive cis-elements. (C) Stress related cis-elements. (D) Plant growth related cis-elements. [file Image_2.tif]
